# Supplementary material for: Dynamic remodeling of the pancreas immune landscape in obesity
Source: Res Sq. 2025 Aug 27:rs.3.rs-7123997. Preprint. [Version 1] doi: 10.21203/rs.3.rs-7123997/v1 (PMC12408010; doi:10.21203/rs.3.rs-7123997/v1)
Supplement: 1 [file NIHPPRS7123997V1-supplement-1.pdf]

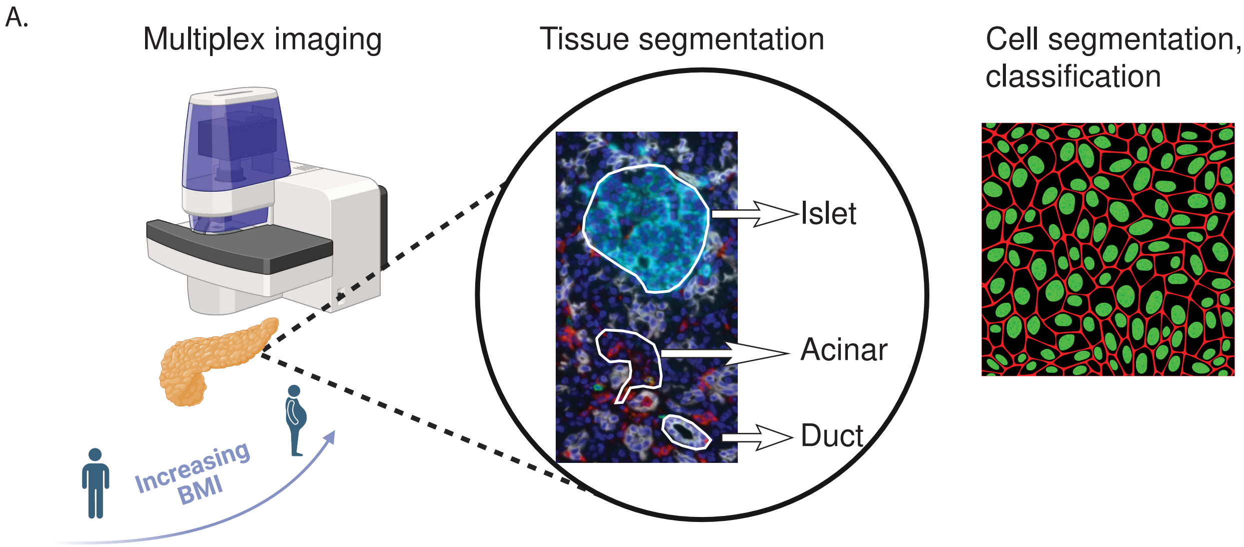
A.
Multiplex imaging
Tissue segmentation
Cell segmentation,
classification
Islet
Acinar
Duct
Increasing
BMI

Figure S1

Figure S1 related to Figure 1. Schematic for immune lineage analysis in exocrine and endocrine pancreas of obese and non-obese organ donors. (A) Multiplex imaging was performed on obese and non-obese organ donors, the cytokeratin 19 (CK19) and chromogranin markers were used to segment tissue into ductal, acinar and endocrine compartments, followed by cell segmentation and classification.

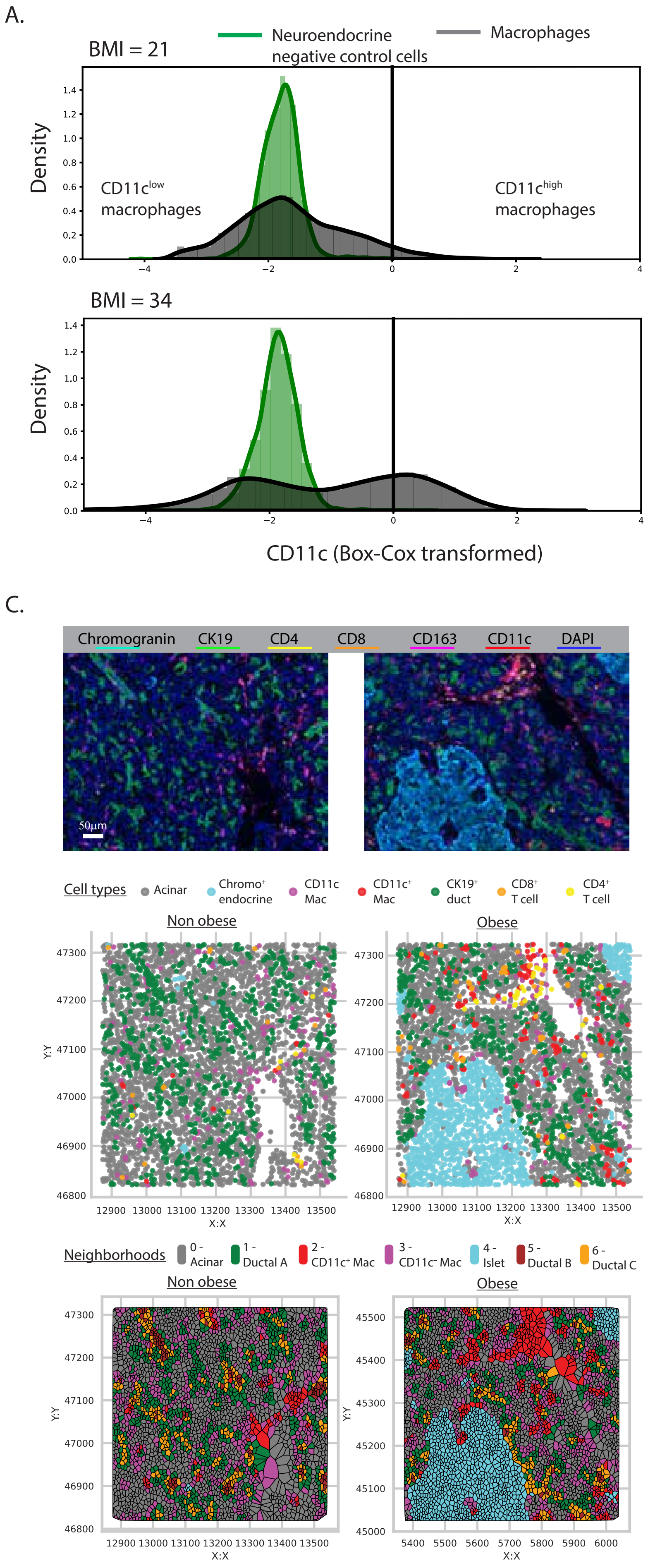
A.
BMI = 21
Neuroendocrine negative control cells
Macrophages
Density
CD11c^low macrophages
CD11c^high macrophages
BMI = 34
Density
CD11c (Box-Cox transformed)
B.
Proportion CD11c^high macrophages
BMI
20.89
21.42
23.31
23.63
24.1
24.69
30.3
31.39
33.98
36.29
36.92
37.07
39.54
41.73
42.43
43.93
Ductal
Acinar
Islet
0.17 0.041 0.021
0.29 0.12 0.015
0.12 0.057 0.42
0.17 0.041 0
0.54 0.14 0.14
0.65 0.54 0.45
0.29 0.22 0.089
0.41 0.2 0.075
0.24 0.1 0.021
0.61 0.31 0.14
0.56 0.36 1
0.44 0.11 0.067
0.5 0.18 0.1
0.51 0.39 0.6
0.47 0.28 0.37
0.41 0.25 0.056
0.41 0.11 0.18
0.75 0.23 0.044
0.23 0.13 0.033
0.19 0.088 0.15
0.62 0.18 0.067
0.03 0.0059 0.018
0.33 0.045 0.098
0.7 0.19 0.18
0.96 0.91 0.86
0.56 0.35 0.28
0.12 0.14 0.075
0.48 0.21 0.17
0.68 0.59 0.21
0.78 0.56 0.42
0.59 0.16 0.19
0.71 0.49 0.11
C.
Chromogranin
CK19
CD4
CD8
CD163
CD11c
DAPI
50µm
Cell types
Acinar
Chromo+ endocrine
CD11c− Mac
CD11c+ Mac
CK19+ duct
CD8+ T cell
CD4+ T cell
Non obese
Obese
Y:Y
X:X
Neighborhoods
0 - Acinar
1 - Ductal A
2 - CD11c+ Mac
3 - CD11c− Mac
4 - Islet
5 - Ductal B
6 - Ductal C
Non obese
Obese
Y:Y
X:X

Figure S2

Figure S2. Related to Figure 2. Macrophage classification and neighborhood analysis strategy. (A) Box-Cox transformed density plots illustrating the gating strategy to classify CD11c^{high} and CD11c^{low} macrophage subsets with comparison to CD11c^{-} neuroendocrine cells. (B) Heatmap showing the ratio of CD11c^{high} macrophages to the total macrophage population across pancreatic compartments in donors with BMI between 21 – 47 kg/m^2. (C) The cell and neighborhood classifications are depicted. Multiplex fluorescence images of pancreatic tissue sections from lean (left) and obese (right) donors, showing CD163$^+$ macrophages (red), CK19$^+$ ducts (white), chromogranin$^+$ islets (cyan), CD4$^+$ T-cells (yellow), CD8$^+$ T-cells (green), and DAPI-stained nuclei (blue) (top). Based on the multiplex images, the classified cell types (CT) from lean (left) and obese (right) donors are plotted on a Cartesian plane (middle). Voronoi diagrams of these images depicting spatial clustering of cell types into seven neighborhoods (NHs) for lean (left) and obese (right) donors, plotted on a Cartesian plane (bottom).

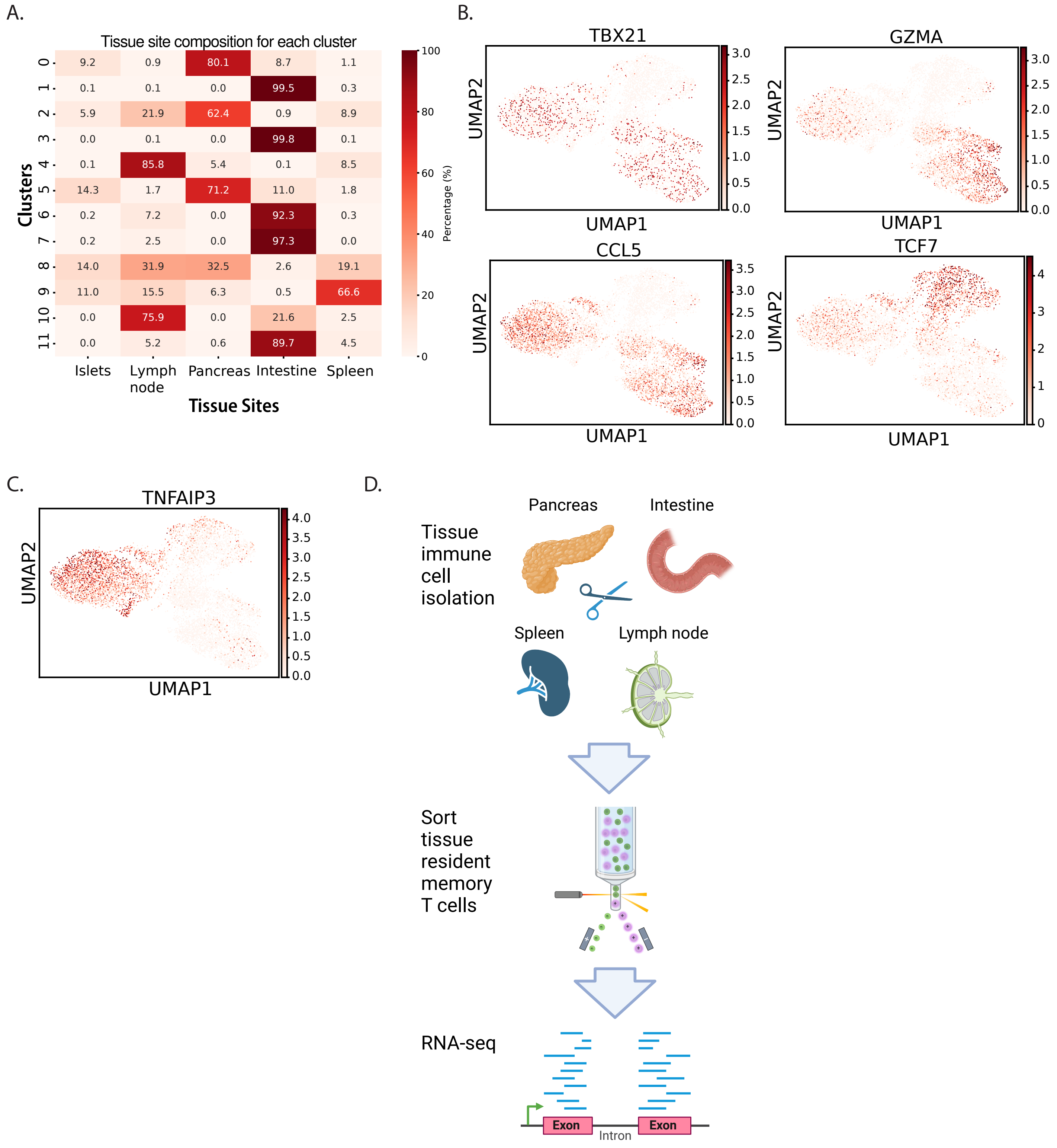
A.
Tissue site composition for each cluster
Clusters
0 9.2 0.9 80.1 8.7 1.1
1 0.1 0.1 0.0 99.5 0.3
2 5.9 21.9 62.4 0.9 8.9
3 0.0 0.1 0.0 99.8 0.1
4 0.1 85.8 5.4 0.1 8.5
5 14.3 1.7 71.2 11.0 1.8
6 0.2 7.2 0.0 92.3 0.3
7 0.2 2.5 0.0 97.3 0.0
8 14.0 31.9 32.5 2.6 19.1
9 11.0 15.5 6.3 0.5 66.6
10 0.0 75.9 0.0 21.6 2.5
11 0.0 5.2 0.6 89.7 4.5
Islets
Lymph node
Pancreas
Intestine
Spleen
Tissue Sites
Percentage (%)
0
20
40
60
80
100
B.
TBX21
GZMA
CCL5
TCF7
UMAP1
UMAP2
C.
TNFAIP3
UMAP1
UMAP2
D.
Tissue immune cell isolation
Pancreas
Intestine
Spleen
Lymph node
Sort tissue resident memory T cells
RNA-seq
Exon
Intron
Exon

Figure S3

Figure S3 related to Figure 3 Quantification of tissue enrichment for each T-cell cluster in the single cell sequencing dataset, feature plots of selected marker genes for the clusters; and a schematic of the bulk RNA-seq workflow comparing CD8 TRM from obese and non-obese donors. (A) The percentage of cells from each tissue - pancreas, islets, lymph node, intestine and spleen – is shown for each cluster identified from the UMAP embeddings of the CITE-seq dataset. The color scaling is across the rows. (B) Normalized values for the indicated transcripts are depicted on the scaled feature plots including markers of effector memory (*TBX21*, *GZMA*, *CCL5*) and naïve/central memory T-cells (*TCF7*). (C) Feature plot of the negative regulator of inflammation, *TNFAIP3*. (D) Schematic showing the workflow for sorting and bulk RNAseq of CD8 TRM from multiple tissues of obese and non-obese organ donors.

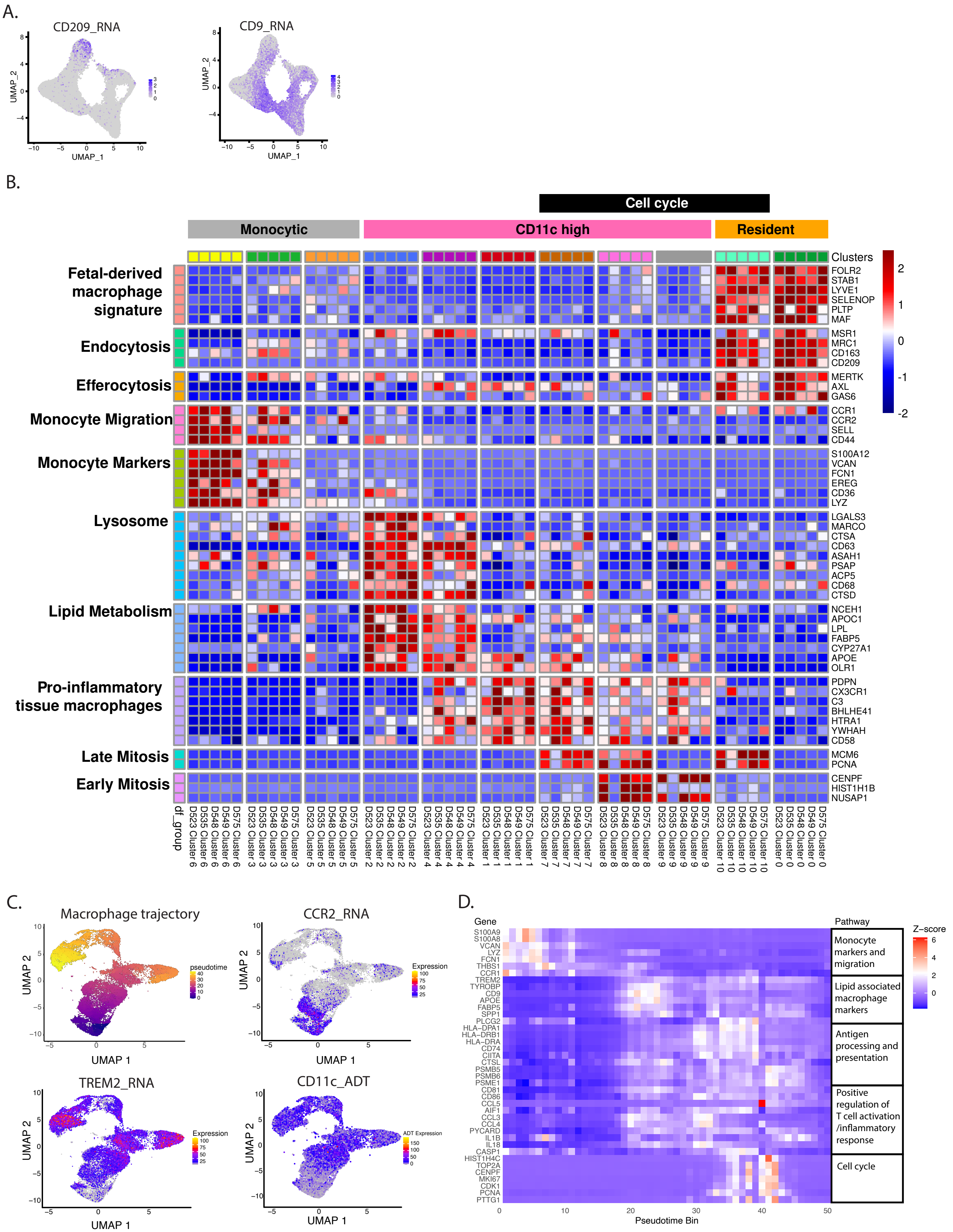
A.
CD209_RNA
CD9_RNA
UMAP_1
UMAP_2
B.
Cell cycle
Monocytic
CD11c high
Resident
Clusters
Fetal-derived macrophage signature
Endocytosis
Efferocytosis
Monocyte Migration
Monocyte Markers
Lysosome
Lipid Metabolism
Pro-inflammatory tissue macrophages
Late Mitosis
Early Mitosis
FOLR2
STAB1
LYVE1
SELENOP
PLTP
MAF
MSR1
MRC1
CD163
CD209
MERTK
AXL
GAS6
CCR1
CCR2
SELL
CD44
S100A12
VCAN
FCN1
EREG
CD36
LYZ
LGALS3
MARCO
CTSA
CD63
ASAH1
PSAP
ACP5
CD68
CTSD
NCEH1
APOC1
LPL
FABP5
CYP27A1
APOE
OLR1
PDPN
CX3CR1
C3
BHLHE41
HTRA1
YWHAH
CD58
MCM6
PCNA
CENPF
HIST1H1B
NUSAP1
df_group
D523 Cluster 6
D535 Cluster 6
D548 Cluster 6
D549 Cluster 6
D575 Cluster 6
D523 Cluster 3
D535 Cluster 3
D548 Cluster 3
D549 Cluster 3
D575 Cluster 3
D523 Cluster 5
D535 Cluster 5
D548 Cluster 5
D549 Cluster 5
D575 Cluster 5
D523 Cluster 2
D535 Cluster 2
D548 Cluster 2
D549 Cluster 2
D575 Cluster 2
D523 Cluster 4
D535 Cluster 4
D548 Cluster 4
D549 Cluster 4
D575 Cluster 4
D523 Cluster 1
D535 Cluster 1
D548 Cluster 1
D549 Cluster 1
D575 Cluster 1
D523 Cluster 7
D535 Cluster 7
D548 Cluster 7
D549 Cluster 7
D575 Cluster 7
D523 Cluster 8
D535 Cluster 8
D548 Cluster 8
D549 Cluster 8
D575 Cluster 8
D523 Cluster 9
D535 Cluster 9
D548 Cluster 9
D549 Cluster 9
D575 Cluster 9
D523 Cluster 10
D535 Cluster 10
D548 Cluster 10
D549 Cluster 10
D575 Cluster 10
D523 Cluster 0
D535 Cluster 0
D548 Cluster 0
D549 Cluster 0
D575 Cluster 0
C.
Macrophage trajectory
pseudotime
CCR2_RNA
Expression
TREM2_RNA
CD11c_ADT
ADT Expression
UMAP 1
UMAP 2
D.
Gene
Pathway
Z-score
S100A9
S100A8
VCAN
LYZ
FCN1
THBS1
CCR1
TREM2
TYROBP
CD9
APOE
FABP5
SPP1
PLCG2
HLA−DPA1
HLA−DRB1
HLA−DRA
CD74
CIITA
CTSL
PSMB5
PSMB6
PSME1
CD81
CD86
CCL5
AIF1
CCL3
CCL4
PYCARD
IL1B
IL18
CASP1
HIST1H4C
TOP2A
CENPF
MKI67
CDK1
PCNA
PTTG1
Monocyte markers and migration
Lipid associated macrophage markers
Antigen processing and presentation
Positive regulation of T cell activation /inflammatory response
Cell cycle
Pseudotime Bin

Figure S4

Figure S4. Related to Figure 4. Cluster marker visualization across individual donors and pseudo-time trajectory model of macrophage gene expression. (A) Feature plots show the normalized expression of the *CD209* (left) and *CD9* (right) with expression level corresponding to purple color intensity for individual cells on the UMAP. (B) Shown is a heatmap of normalized RNA expression for marker genes of the macrophage clusters scaled by row and averaged for each individual donor and cluster. The cluster and donor numbers are shown along the bottom with corresponding cluster colors indicated along the top with macrophage classifications based on phenotype and gene expression. The genes are organized by function (left). (C) Series of Uniform Manifold Approximation and Projection (UMAP) plots showing the inferred pseudo-time trajectory of macrophage differentiation (clusters #1-9) and associated expression of key markers that are significantly associated with trajectory progression. The UMAP layout is derived from transcriptional profiles, with cells colored by the following: Pseudo-time progression along the differentiation pathway (top left); *CCR2* RNA (top right); *TREM2* RNA expression (bottom left); CD11c protein expression as measured by antibody-derived tag (bottom right). Color intensity corresponds to the respective feature's scaled and normalized value. (D) Heatmap of Z-score normalized gene expression (rows) for key marker genes that are significantly associated with pseudo temporal trajectory progression. The pseudo time bins are shown along the bottom and genes organized by function based on gene set enrichment analysis are shown along the right side.

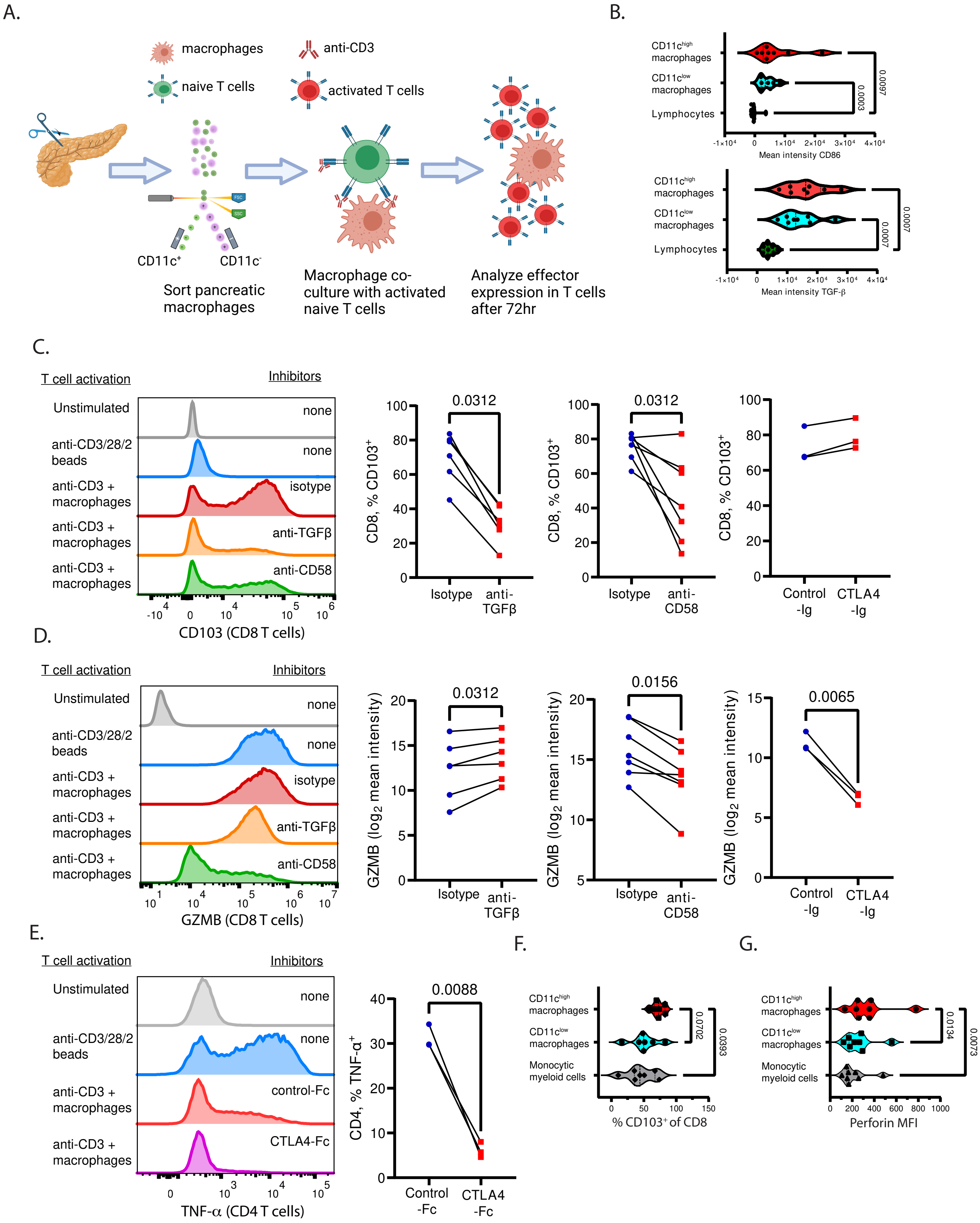

A.
macrophages
anti-CD3
naive T cells
activated T cells
CD11c+
CD11c-
Sort pancreatic macrophages
Macrophage co-culture with activated naive T cells
Analyze effector expression in T cells after 72hr
B.
Mean intensity CD86
Mean intensity TGF-β
Lymphocytes
C.
T cell activation
Inhibitors
Unstimulated
anti-CD3/28/2 beads
anti-CD3 + macrophages
none
isotype
anti-TGFβ
anti-CD58
CD103 (CD8 T cells)
0.0312
Isotype
anti-TGFβ
anti-CD58
Control -Ig
CTLA4 -Ig
D.
GZMB (CD8 T cells)
0.0156
0.0065
E.
control-Fc
CTLA4-Fc
TNF-α (CD4 T cells)
0.0088
Control -Fc
CTLA4 -Fc
F.
Monocytic myeloid cells
% CD103+ of CD8
G.
Perforin MFI

Figure S5

Figure S5 related to Figure 5. The impact of pancreatic macrophage-derived factors on T-cell function. (A) Schematic of the macrophage/T-cell co-culture system used to define how the different pancreatic macrophage subsets influence effector and residency molecule expression by T-cells. (B) Shown are the mean fluorescence values of surface CD86 (top) and intracellular TGF-β (bottom) by flow cytometry from the indicated pancreatic macrophage subsets (n=8). P values were calculated by one-way ANOVA and Holm-Sidak multiple comparison test. (C, D) Representative histograms (left) of CD8 T-cells show CD103 expression (C) and Granzyme B (GZMB) expression (D) under the following conditions: unstimulated (grey); activation with anti-CD3/CD2/CD28 beads alone (blue); activation with monomeric anti-CD3 in pancreatic macrophage co-culture with isotype control (red) and with antibody inhibitors against TGF-β (orange) and CD58 (green). The compiled data (right) show the impact of the indicated inhibitors (TGF-β, n=6; CD58, n=7 and CTLA4-Fc, n=3) on CD8 T-cell expression of CD103 (C) and GZMB (D) after activation and pancreatic macrophage co-culture. (E) Representative histograms (left) of CD4 T-cells show TNF-α expression under the following conditions: unstimulated (grey); activation with anti-CD3/CD2/CD28 beads alone (blue), activation with monomeric anti-CD3 in pancreatic macrophage co-culture with control-Fc (red) and the CD86 inhibitor, CTLA4-Fc (magenta). The compiled data (right) show the effect of CTLA4-Ig compared to control-Fc on CD4 T-cell expression of TNF-α after activation and pancreatic macrophage co-culture (n=3). P values were calculated using a non-parametric paired t-test to determine inhibitor treatment effect versus control. (F, G) Shown are compiled flow cytometry data from naïve T-cells after activation and co-culture with the indicated pancreatic macrophage subsets quantifying the percentages of $CD103^{+}$ CD8 T cells (F) and mean fluorescence intensity of perforin (n=8) (G). P values were calculated by one-way ANOVA and Holm-Sidak multiple comparison test.

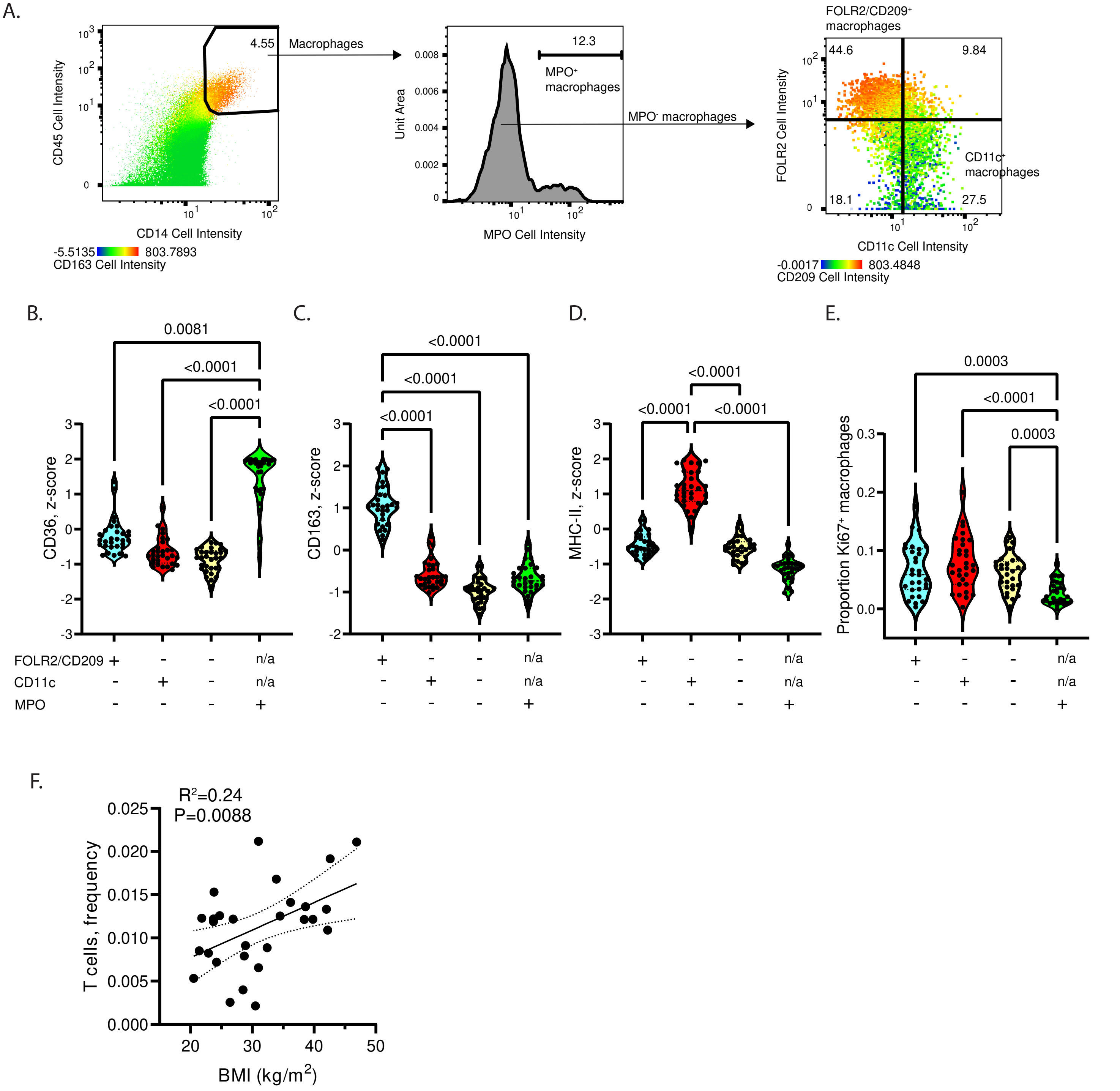
A.
4.55
Macrophages
CD45 Cell Intensity
CD14 Cell Intensity
-5.5135
803.7893
CD163 Cell Intensity
12.3
MPO⁺ macrophages
MPO⁻ macrophages
Unit Area
MPO Cell Intensity
FOLR2/CD209⁺ macrophages
44.6
9.84
18.1
27.5
CD11c⁺ macrophages
FOLR2 Cell Intensity
CD11c Cell Intensity
-0.0017
803.4848
CD209 Cell Intensity
B.
C.
D.
E.
0.0081
<0.0001
<0.0001
CD36, z-score
CD163, z-score
MHC-II, z-score
0.0003
0.0003
Proportion Ki67⁺ macrophages
FOLR2/CD209
CD11c
MPO
n/a
F.
R²=0.24
P=0.0088
T cells, frequency
BMI (kg/m²)

Figure S6

Figure S6. Related to Figure 6. Strategy for macrophage classification based on multiplex imaging of pancreas tissue microarray. (A) Gating strategy for macrophage classification from segmented cells on pancreas tissue microarrays (left), the identification MPO^+ macrophages (middle), and further sub-setting of MPO^- macrophages into FOLR2/$CD209^+$ $CD11c^-$, FOLR2/$CD209^-$ $CD11c^+$ and FOLR2/$CD209^-$ $CD11c^+$ macrophage populations (right). (B-E), Violin plots showing Z score normalized expression of (B) CD36, (C) CD16, (D) MHC-II and (E) the proportion of $KI67^+$ macrophages across the indicated phenotypes (shown below each plot). P values were calculated using one-way ANOVA with Dunn's multiple comparisons testing. (F) The scatterplot of T cells frequency correlated with donor BMI (n=28). The best fit lines, 95% confidence intervals and P values were calculated using simple linear regression analysis.

Table S1: Pancreas Disease Risk Factors Examined with Multivariable Models

	Non-obese N=12	Obese N=20
Continuous variables, median (±SD)		
BMI (kg/m^2)	23.2 (1.4)	37.8 (4.7)
Age (years)	49.3 (12.5)	47.5 (19.3)
HbA1c (%)	5.4 (0.32)	6.9 (2.5)
Categorical variables, n (%)		
Diabetes	0 (0%)	9 (45%)
Heavy alcohol use (2+ drinks/daily)	1 (8.3%)	5 (25%)
Cigarette use (>20 pack years) ever	1 (8.3%)	6 (30%)
Male sex	8 (67%)	9 (45%)
Abbreviations: BMI, body mass index; SD, standard deviation; HbA1c, hemoglobin A1c		

Table S2. Multivariable analysis of pancreas immune cells.

Variable	CD4 T cells*			CD8 T cells			Macrophages		
	Regression Coefficient	SE	P value	Regression Coefficient	SE	P value	Regression Coefficient	SE	P value
**Acinar**									
BMI	0.059	0.017	0.0012	3.63	1.28	0.0083			
Age	n/a	n/a	n/a	1.26	0.66	0.066			
Diabetes	-0.62	0.29	0.039	n/a	n/a	n/a			
Heavy alcohol use	0.59	0.32	0.072	n/a	n/a	n/a			
Smoking	n/a	n/a	n/a	27.53	25.54	0.29			
Male	n/a	n/a	n/a	-35.17	22.07	0.12			
**Ductal**									
BMI				3.79	1.39	0.011			
Age				1.55	0.72	0.039			
Diabetes				n/a	n/a	n/a			
Heavy alcohol use				n/a	n/a	n/a			
Smoking				67.6	27.79	0.022			
Male				-48.84	24.02	0.0519			
**Islet**									
BMI	0.73	0.27	0.010				12.67	3.85	0.0027
Age	n/a	n/a	n/a				n/a	n/a	n/a
Diabetes	-6.165	4.742	0.20				n/a	n/a	n/a
Heavy alcohol use	n/a	n/a	n/a				126.5	76.14	0.11
Smoking	n/a	n/a	n/a				81.64	73.65	0.28
Male	n/a	n/a	n/a				n/a	n/a	n/a

Abbreviations: BMI, body mass index; SE, standard error. *, indicates data are natural log transformed

Results shown are from multiple linear regression models (least squares regression type) in which the outcome variables are CD4 T cell, CD8 T cell and macrophage density within the indicated pancreas tissue compartment. For those analyses that showed significant correlation of BMI with the outcome variable by simple linear regression, multiple linear regression analysis was run with BMI and all the indicated co-variates in an initial model. Only those co variates showing possible association with the outcome variable (P<0.3) were used in the final model. Those variables excluded from the final model due to lack of association with the outcome are indicated (n/a). For each variable the P values reported were calculated using a two-sided test.

Table S3. Pathway and ontology analysis of pancreatic TRM transcriptomic signatures.

Transcripts higher in pancreas-enriched TRM clusters

Term	Adjusted P-value	Odds Ratio	Data set
CTL mediated immune response against target cells	8.99E-04	64.17	Bioplanet 2019
T-Cell Cytotoxic Mediated Cell Death	8.34E-06	31.53	Elsevier Pathway Collection
Binding of chemokines to chemokine receptors	3.35E-04	22.26	Bioplanet 2019
T cell receptor regulation of apoptosis	4.53E-06	6.58	Bioplanet 2019

Transcripts lower in pancreas-enriched TRM clusters

Term	Adjusted P-value	Odds Ratio	Data set
Cellular Response To Corticosteroid Stimulus (GO:0071384)	1.77E-04	33.60	GO Biological Process 2023
Negative Regulation of MAPK Pathway	9.66E-07	18.01	Reactome Pathways 2024
Cortisol in Resolving Inflammation	2.84E-06	17.07	Elsevier Pathway Collection
Negative Regulation Of Intracellular Signal Transduction (GO:1902532)	6.99E-04	4.43	GO Biological Process 2023

Abbreviations: TRM, tissue resident memory T-cell

Results shown are from pathway and ontology analysis of differentially expressed transcripts (adjusted P value cutoff < 0.05, absolute value of $\log_2$ fold change cutoff > 0.3) in the pancreas-enriched CD8 TRM clusters (# 0, 2, 5 comprising 3116 cells) compared to intestine-enriched CD8 TRM clusters (# 1, 3, 7 comprising 2686 cells) and all other T-cells in the dataset (6098 cells). Transcripts found to be differentially expressed in the pancreas-enriched CD8 TRM clusters by both comparisons were entered into separate pathway analyses for transcripts that were found to be higher (top) and lower (bottom) in the pancreas-enriched CD8 TRM. For each pathway or ontology term the adjusted P-value, odds ratio and curated data set are shown.

Table S4. Pathway and ontology analysis of genes from pancreatic TRM that are significantly increased in obesity.

	**Pancreatic TRM**		**Intestine TRM**		**Lymph node/spleen TRM**	
Term (MSigDB Hallmark 2020)	Adjusted P -value	Score	Adjusted P -value	Score	Adjusted P -value	Score
Interferon Gamma Response	1.59E-15	329.65	2.97E-03	39.53	n/a	n/a
Inflammatory Response	1.75E-10	169.46	*ns*	1.07	n/a	n/a
Interferon Alpha Response	3.05E-08	174.88	*ns*	23.24	n/a	n/a
TNF-alpha Signaling via NF-kβ	9.87E-08	98.00	2.12E-04	66.72	n/a	n/a
Allograft Rejection	9.87E-08	98.00	*ns*	8.83	n/a	n/a

Abbreviations: TRM, tissue resident memory T-cell; *ns*, not significant.

Results shown are from pathway and ontology analysis of transcripts (adjusted P value cutoff < 0.05) from sorted TRM that are significantly increased in obese (n=5) compared to non-obese (n=6) organ donors. For each pathway or ontology term the adjusted P-values, and enrichment scores from the indicated curated data set are shown.

Table S5. Multivariable analysis of pancreatic macrophage subsets.

Variable	FOLR2/CD209^{+} macrophages*			CD11c^{+} macrophages*		
	Regression Coefficient	SE	P value	Regression Coefficient	SE	P value
BMI	-0.0093	0.0037	0.020	0.0044	0.0013	0.0028
Age	n/a	n/a	n/a	n/a	n/a	n/a
Diabetes	0.091	0.11	0.40	-0.058	0.040	0.16
Smoking	n/a	n/a	n/a	n/a	n/a	n/a
Male	-0.078	0.058	0.20	n/a	n/a	n/a
TMA1 (ref TMA2)	-0.26	0.071	0.0012	0.16	0.023	0.0001
TMA3 (ref TMA2)	-0.081	0.059	0.18	0.054	0.022	0.025

Abbreviations: BMI, body mass index; SE, standard error; TMA, tissue micro-array. *, indicates data are natural log transformed

Results shown are from multiple linear regression models (least squares regression type) in which the outcome variables are the proportions of the indicated macrophage subsets of all macrophages. The FOLR2/CD209^{+} subset has the phenotype, CD45^{+}CD14^{+}MPO^{-}CD11c^{-} and FOLR2/CD209^{+}. The CD11c+ subset has the phenotype, CD45^{+}CD14^{+}MPO^{-}CD11c^{+} and FOLR2/CD209^{-}. Multiple linear regression analysis was run using BMI and all the indicated co-variates in an initial model. In addition to the biological co-variates, the analysis corrected for TMA batch. Only those co variates showing possible association with the outcome variable ($P \leq 0.3$) were used in the final model. Those variables excluded from the final model due to lack of association with the outcome are indicated (n/a). For each variable the P values reported were calculated using a two-sided test.

Table S6. Multivariable analysis of Gal-3^{+} pancreatic epithelium and T-cells.

Variable	Gal-3^{+} epithelial cells*			T-cells		
	Regression Coefficient	SE	P value	Regression Coefficient	SE	P value
BMI	-0.033	0.013	0.019	0.00045	0.00015	0.0078
Age	0.0067	0.0053	0.22	0.000072	0.000053	0.19
Diabetes	n/a	n/a	n/a	-0.0053	0.0043	0.24
Smoking	n/a	n/a	n/a	0.0039	0.0039	0.32
Male	0.20	0.21	0.37	0.0042	0.0021	0.057
TMA1 (ref TMA2)	-1.7	0.26	0.0001	-0.0055	0.0024	0.035
TMA3 (ref TMA2)	-2.2	0.21	0.0001	n/a	n/a	n/a

Abbreviations: BMI, body mass index; SE, standard error; TMA, tissue micro-array. *, indicates data are natural log transformed

Results shown are from multiple linear regression models (least squares regression type) in which the outcome variables are the proportions of the indicated cell type of all cells in each sample. The Gal-3^{+} subset shows co-expression of E-cadherin with Gal-3 including the E-cadherin^{+}cytokeratin^{+} ductal cells and E-cadherin^{+}cytokeratin^{-} acinar cells. The T-cells are the sum of the CD3^{+}CD4^{+} and CD3^{+}CD8^{+} cells. Multiple linear regression analysis was run using BMI and all the indicated co-variates in an initial model. In addition to the biological co-variates the analysis corrected for TMA batch. Only those co variates showing possible association with the outcome variable (P≤0.3) were used in the final model. Those variables excluded from the final model due to lack of association with the outcome are indicated (n/a). For each variable the P values reported were calculated using a two-sided test.
